# From Cell Architecture to Mitochondrial Signaling: Role of Intermediate Filaments in Health, Aging, and Disease

**DOI:** 10.3390/ijms26031100

**Published:** 2025-01-27

**Authors:** Emanuele Marzetti, Rosa Di Lorenzo, Riccardo Calvani, Vito Pesce, Francesco Landi, Hélio José Coelho-Júnior, Anna Picca

**Affiliations:** 1 Fondazione Policlinico Universitario “Agostino Gemelli” IRCCS, L.go A. Gemelli 8, 00168 Rome, Italy; emanuele.marzetti@policlinicogemelli.it (E.M.); riccardo.calvani@unicatt.it (R.C.); francesco.landi@unicatt.it (F.L.); coelhojunior@hotmail.com.br (H.J.C.-J.); 2 Department of Geriatrics, Orthopedics and Rheumatology, Università Cattolica del Sacro Cuore, L.go F. Vito 1, 00168 Rome, Italy; 3 Department of Biosciences, Biotechnologies and Environment, Università degli Studi di Bari Aldo Moro, Via Edoardo Orabona 4, 70125 Bari, Italy; rosa.dilorenzo@uniba.it (R.D.L.); vito.pesce@uniba.it (V.P.); 4 Department of Medicine and Surgery, LUM University, Str. Statale 100, 70010 Casamassima, Italy

**Keywords:** axonal transport, cell architecture, cell quality, cytoskeleton, mitochondrial quality, muscle aging, neurodegeneration, neurofilaments, sarcomere, vimentin

## Abstract

The coordination of cytoskeletal proteins shapes cell architectures and functions. Age-related changes in cellular mechanical properties have been linked to decreased cellular and tissue dysfunction. Studies have also found a relationship between mitochondrial function and the cytoskeleton. Cytoskeleton inhibitors impact mitochondrial quality and function, including motility and morphology, membrane potential, and respiration. The regulatory properties of the cytoskeleton on mitochondrial functions are involved in the pathogenesis of several diseases. Disassembly of the axon’s cytoskeleton and the release of neurofilament fragments have been documented during neurodegeneration. However, these changes can also be related to mitochondrial impairments, spanning from reduced mitochondrial quality to altered bioenergetics. Herein, we discuss recent research highlighting some of the pathophysiological roles of cytoskeleton disassembly in aging, neurodegeneration, and neuromuscular diseases, with a focus on studies that explored the relationship between intermediate filaments and mitochondrial signaling as relevant contributors to cellular health and disease.

## 1. Introduction

Cell architecture and function rely on the efficient coordination of dynamic cytoskeletal proteins (i.e., actin microfilaments, microtubules, and intermediate filaments) that organize and rapidly adapt their network upon internal and external stimuli [1].

Age-associated changes in cellular mechanical properties, including greater stiffness and reduced elasticity and strength, have been described in several cell types, including vascular endothelial cells, cardiomyocytes, skeletal muscle cells, and immune cells [2]. These changes usually lead to decreased cellular sensitivity and compromised tissue responsiveness [2].

During aging, a high collagen deposition and declines in elastin have been described in vascular muscle cells [3,4], which reduce the contraction and dilation ability of blood vessels [3,4,5,6]. Structural changes in cell mechanics have also been reported in aged cardiomyocytes with higher crosslinking density of extracellular matrix and significant increases in cytoplasmic viscosity and cell adhesion of profibrotic fibroblasts [7]. Altogether, these changes reduce cardiac tissue contraction and relaxation as well as increase the risk of developing cardiovascular disease [7].

Age-related deteriorations of the musculoskeletal system are also well documented. These include reduced bone mineral density and loss of skeletal muscle mass and strength, which predispose to a higher risk of osteoporosis, fractures, sarcopenia, and physical frailty [8,9]. Cellular and ultrastructural changes in skeletal myofibers and mononucleated muscle stem cells/satellite cells (MSCs) have also been described in sarcopenia [10]. Of note, sex-associated variations in such parameters have been reported and indicated as relevant biological contributors to the frailty status [11]. Age-dependent changes in the osteocyte cortical diameter, thickness, density, and porosities have been described in preclinical models [12,13], with osteocyte mechanical degeneration implicated in bone mass loss, altered mechanosensitive calcium signaling, and impaired bone mechanosensitive responses [12,14,15].

Declines in immune cell functions with aging have also been associated with changes in cell mechanics. An age-related deterioration of the lymphocyte cytoskeleton has been described and related to increased stiffness in cells from old donors compared with young counterparts [16]. Moreover, reduced membrane deformability of red blood cells with aging has been associated with diminished cell function [17].

Most research has pinpointed the role of structural and architectural cytoskeletal protein changes; however, emerging studies highlight a relationship between mitochondrial features and signaling (e.g., motility and fission/fusion dynamics) and the activity of the cytoskeleton [18,19,20].

Mitochondrial quality and function decline with aging, leading to the heightened generation of reactive oxygen species (ROS) that contribute to cellular senescence and aging [21,22]. Preclinical data indicate a close relationship between mitochondrial biogenesis and antioxidant activity [23]. In the setting of increased ROS production, oxidizing reactions are triggered against cellular components, inflicting damage to nucleic acids, proteins, and membrane lipids [24,25]. These modifications impact the function, conformation, and subcellular localization of these macromolecules and interfere with biomolecular interactions and overall cellular function [25].

Experiments in cell culture systems have shown that cytoskeleton inhibitors impact mitochondrial quality and function, including motility and morphology, membrane potential, and respiration [19]. The regulatory effect of the cytoskeleton on mitochondrial bioenergetics is even more relevant if considering its implications in several diseases. Growing evidence reports the disassembly of an axon’s structure and release of neurofilament (Nfl) fragments at the systemic level in individuals with neurodegenerative diseases [26,27,28]. Circulating levels of Nfls may, therefore, mirror their abnormal accumulation and assembly in cell bodies and the axon of motor neurons, which can interfere with normal axonal transport. However, these changes can also be related to mitochondrial impairments spanning from reduced mitochondrial quality to altered bioenergetics [29,30].

Herein, we discuss recent research highlighting some of the pathophysiological roles of cytoskeleton disassembly in aging and pathological conditions with a focus on studies identifying a relationship between intermediate filaments (IFs) and mitochondrial signaling as relevant contributors to cellular health and disease.

## 2. Intermediate Filaments in the Economy of the Cytoskeleton

The cytoskeleton is the cellular scaffold composed of a dynamic set of proteins, including actin microfilaments, microtubules, and IFs [1]. Of these, microtubules and acting microfilaments exist in a few isoforms and share high degrees of similarity across eukaryotes.

IFs are a large family of elongated proteins involved in maintaining structural and mechanical cell’s integrity [31]. IFs contain a central conserved helix–loop–helix domain of around 40 repeated motifs of seven amino acids forming extended spiral structures with other monomers. Within a filament, a pair of parallel dimers associate with an antiparallel pair, forming a staggered tetramer. Several tetramers join laterally to form the filament, which comprises eight parallel protofilaments composed of tetramers. This large number of stacked polypeptides aligned together, with the strong lateral hydrophobic interactions typical of proteins with a spiral structure, confers IFs rope-like characteristics without polarity. Remodeling of the IF network accompanies events that require a dynamic reorganization of the cell, such as division, migration, and differentiation [31].

**Figure 1:**
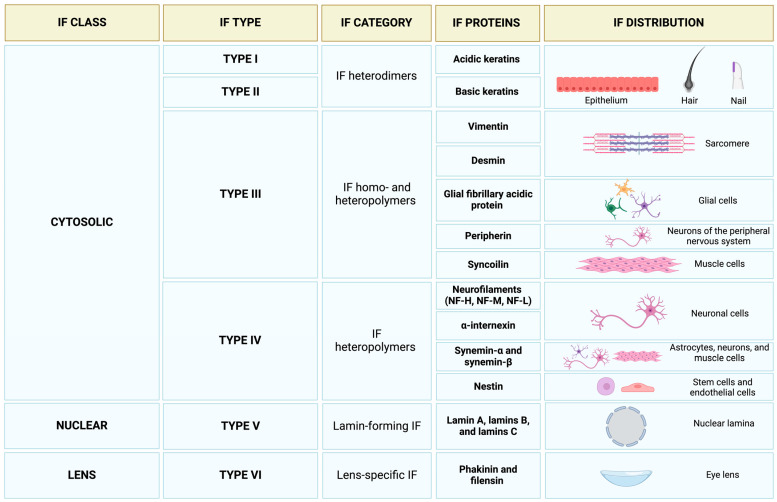
Schematic classification of intermediate filaments and protein types. Abbreviations: IF, intermediate filament; NF-H, high molecular weight neurofilaments; NF-L, low molecular weight neurofilaments; NF-M, middle molecular weight neurofilaments. Created in https://BioRender.com.

Cytoplasmic IFs can be grouped into three main classes, including types I and II keratin filaments of epithelial cells, type III vimentin and vimentin-like filaments of connective tissue, muscle, and neuroglial cells, and type IV Nfls of neurons (Figure 1).

Types I and II keratin filaments include acidic and basic keratins assembling into type I/type II heterodimers. Such keratin pairs can be found in single-layer epithelium as simple keratins, in multilayer epithelium as epidermal keratins, and in nails and hair as structural keratins [32]. Type III filament proteins include vimentin and vimentin-like members (i.e., desmin, glial fibrillary acidic protein (GFAP), peripherin, and syncoilin), forming homo- and heteropolymers [33,34]. Of these proteins, GFAP is expressed by glial cells, while syncoilin can be found in muscle cells [35]. Peripherin is expressed by neurons of the peripheral nervous system and participates in the assembly of Nfls with type IV IF proteins. These latter include high molecular weight Nfl (NF-H, 200 kDa), middle molecular weight Nfl (NF-M, 160 kDa), low molecular weight Nfl (NF-L, 68 kDa), and α-internexin in neurons [36]. Furthermore, synemin-α and synemin-β are found in astrocytes, neurons, and muscle cells, while nestin is expressed by stem and endothelial cells [36,37,38]. Keratins and vimentin are building blocks of the cell cortex [39,40]. These filaments are involved in the organization of a trans-cellular network that interconnects the plasma membrane and the nuclear compartment of individual cells and tissues [41,42,43].

IFs are also an integral part of the nucleus with type V intranuclear filament proteins. This class includes six proteins called lamin A, lamins B, B2, and B3, and lamin C1 and C2 encoded by the alternative splicing of LMNA, LMNB1, and LMNB2 genes [44,45] (Figure 1). IFs form tridimensional layers of lamins in the nucleus, the so-called nuclear lamins, and support and shape the nuclear envelope while offering anchoring platforms for chromosomes to guarantee nuclear and genome integrity [46]. Finally, an additional class of IFs is that of type VI filaments composed of phakinin and filensin proteins. These are expressed in eye lenses with structures differing from canonical IF proteins [47,48] (Figure 1).

While this highly organized network structure emphasizes the mechanosensory roles of IFs, these filaments serve other relevant functions, such as the detection of stress signals, including oxidative stress [49].

## 3. Neurofilaments and Neurodegeneration

Nfls are found in high concentrations along neuronal axons in vertebrates as three subtypes (NF-L, NF-M, and NF-H) that co-assemble in vivo and can form heteropolymers with α-internexin in the central nervous system and with peripherin in the peripheral nervous system [50,51]. Nfl proteins are expressed in different phases of neuronal growth and development: NF-L with peripherin or α-internexin during neuronal differentiation, NF-M concomitantly with axonal elongation, and NF-H together with radial outgrowth and myelination [52,53]. Nfl proteins have an amino-terminal “head” domain for the initiation of Nfl assembly, a central α-helical “rod” domain requested for assembly and integrity, and a variable length carboxy-terminal “tail” domain highly subjected to phosphorylation for its richness in lysine–serine–proline (KSP) motifs [54]. The NF-H and NF-M proteins have long C-terminal tail domains that bind to neighboring filaments, generating aligned arrays with uniform spacing between filaments. The formation and activity of Nfls are regulated by post-transcriptional and post-translational modifiers. This enables the creation of a stable but elastic filamentous structural scaffold within the axon that promotes the local expression of Nfl proteins and the necessary dynamic responses to local demands, stimuli, and possible damages [55]. During axonal growth, new Nfl subunits are incorporated along the axon length in a dynamic process involving the addition of subunits along the entire length of the filament, as well as at its ends. After the axon elongation and connection with its target cell are completed, the axonal diameter can grow up to five times. The expression level of Nfl genes appears to directly control the diameter of the axon, which in turn influences the speed of transmission of electrical signals [52,56]. Nfls also intervene in microtubule dynamics, organelle distribution, and synaptic neurotransmission [55]. Furthermore, Nfls provide strength and stability to the long cellular structures of neurons. Proteasomal and phagocytic pathways are responsible for the degradation of Nfls proteins [57]. Their active release can occur via exosomes [58,59], but the loss of neuronal membrane integrity can favor their passive delivery. The entry of Nfl fragments into the peripheral circulation occurs via drainage along the basement of capillary and arterial membranes [60], carriage toward cervical and/or lumbar lymph nodes, and eventual delivery into the bloodstream. In the setting of axonal damage during aging or in diseases of the nervous system, Nfl fragments can be found in the blood and cerebrospinal fluid (CSF), serving as markers of a neuro-axonal injury [61]. Sensitive assays for measuring Nfls in the CSF are available [62]. NfLs are also found in plasma and serum, but their quantification has long been hampered by the low concentrations (pg/mL), especially in plasma, which is undetectable by many commercial ELISA assays. Novel ultrasensitive techniques have, therefore, been developed (e.g., SiMoA technology, Quanterix) [62], but their implementation in large-scale studies is hampered by their complexity and the need for trained personnel. The advent of recent automated ELISA assays (e.g., ProteinSimple, part of Bio-Techne) has partially addressed this gap [63]. However, head-to-head comparisons between the two methodologies, while confirming their efficiency, indicate that the interpretation of results requires a critical appraisal and knowledge of the specific assay used [63].

Older adults show high levels of NF-L in the CSF that increase with age [64]. Several genes involved in the dynamics of Nfls are known, including GAN (Gigaxonin) encoding Gigaxonin-E3 ubiquitin ligase, TRIM2 (Tripartite Motif Containing 2) encoding Trim2-E3 ligase, KIF5A (Kinesin Family Member 5A) encoding Kinesin-5A, SACS (Sacsin Molecular Chaperone) encoding Sacsin, and HSPB1 (Heat Shock Protein Family B (small) Member 1) encoding the HSPB1 chaperone, whose mutations alter neuronal homeostasis and functionality. However, the role of Nfl aggregation in neurodegenerative diseases remains unclear [53]. High blood/CSF levels of NF-L have been associated with accelerated cognitive decline and/or neurodegeneration in Alzheimer’s disease, although a clear understanding of the relationship between neuronal damage and functional decline is missing [65,66]. Of the various neurodegenerative disorders in which abnormal accumulation, metabolism, and organization of Nfl proteins have been reported, amyotrophic lateral sclerosis (ALS), Parkinson’s disease (PD), and Charcot–Marie–Tooth (CMT) disease are among the best characterized [26,27,28] and will be discussed in the next sections.

### 3.1. Amyotrophic Lateral Sclerosis

ALS is a rare neurodegenerative disease with an incidence of 0.6 to 3.8 cases per 100,000 people every year and a mean age of onset between 51 and 66 years [67]. ALS is characterized by upper and lower motor neuron (MN) dysfunction at the level of the bulbar, cervical, thoracic, and/or lumbar regions of the spinal cord [68]. Axon degeneration leads to weakness, muscle atrophy, paralysis, and, ultimately, fatal respiratory failure [69].

The pathogenic mechanisms of the disease are unclear. However, mitochondrial dysfunction and oxidative stress have been listed among the biological contributors, along with oligodendrocyte dysfunction, cytoskeletal disturbances, and defects in axonal transport [70]. Experiments in ALS mice engineered with mutations in the genes encoding the transactive response DNA-binding protein (TARDBP) and proteins fused-in-sarcoma (FUS) have demonstrated that axonal transport deficits are prodromal features of the disease and contribute to motor dysfunction and loss of neuromuscular integrity [71]. Defects in axonal transport and cytoskeleton in ALS patients are recognizable by the swelling of the axon’s initial segment (AIS) on MNs (i.e., the region of action potential initiation), within which lysosomes, vesicles, mitochondria, and IFs, including Nfls, accrue [72]. The accumulation of Nfls in MNs is a histopathological trait of ALS [73]. However, mutations in the NF-L gene (NEFL) are not the primary cause of ALS. In a single patient with sporadic ALS, a rare polymorphism in the tail domain of NEFL has been identified, indicating the existence of benign (silent) variants or that the pathogenicity is linked to other genetic factors. Two NF-M gene (NEFM) polymorphisms that increase the risk of ALS have been found in another patient with sporadic ALS and in a patient with familial ALS [74]. Mutations in the head, rod, and tail protein domains of the NF-H gene (NEFH) have all been connected to ALS. In particular, deletions or insertions in the tail region can modify the protein phosphorylation domain with important consequences on Nfl maintenance, assembly, and transport [75,76,77,78,79]. Kinases are also responsible for controlling the integrity of the cytoskeletal structure. Protein kinase N1 (PKN1) phosphorylates the rod-head domain of Nfl proteins. A high concentration of active PKN1 blocks the assembly of Nfls and their transport along the axons, causing the aggregation of Nfls in the soma [80,81]. An imbalance between kinase and phosphatase activities also contributes to ALS [82]. Lefebvre-Omar et al. [73] studied the impact of Nfl accumulation on MN viability, comparing MNs derived from induced pluripotent stem cells (iPSCs) from patients with mutations in the chromosome 9 open reading frame 72 (C9orf72), superoxide dismutase 1 (SOD1), and TARDBP genes, all of which involved in ALS onset. In all mutant MNs, NF-Ls rapidly accumulate in the soma, while phosphorylated NF-Ms/Hs accumulate in the axonal proximal regions of only C9orf72 and SOD1 MNs, accompanied by excitability abnormalities, causing impairment of AIS integrity [73]. An aberrant phosphorylation of NF-M and NF-H side arms involves the activity of p38 protein kinases [83]. Using iPSC from ALS patients with a mutation in the SOD1 gene, it was observed that the aggregation of Nfls in spinal MNs was caused by an altered protein proportion of the Nfl subunits, which led to neuronal degeneration in the absence of glia [84]. Currently, serum and CSF Nfls are the most used biomarkers of ALS also in the clinical setting [85]. Serum NF-L concentrations allow ALS patients to be distinguished from healthy individuals and provide information on survival [86]. However, Rossi et al. [87] have demonstrated that levels of NF-L and p-NF-H in the CSF of ALS patients are comparable to those of patients with significant neuronal cell death/axonal damage due to acute/subacute inflammatory diseases or tumors, while they are significantly increased relative to patients with neurological disorders not specifically related to progressive neuronal cell death/axonal damage or acute inflammation. Hence, NF-L and p-NF-H serve as indicators of neuronal degeneration and death, especially in cases in which the disease (e.g., ALS) has a rapid evolution, but they cannot be considered disease-specific biomarkers [87]. Nevertheless, the monitoring of serum NF-L levels has shown to be useful for evaluating the response of patients with SOD1-ALS to treatment with the antisense oligonucleotide tofersen [88]. The determination of circulating anti-NF-L antibodies has proven useful for prognostication [89]. However, a causal link between Nfl pathology and ALS has not yet been fully established. Moreover, in patients with ALS, the blockade of axonal transportation is further aggravated by mitochondrial dysfunction, including dysregulation of mitochondrial proteins, increased ROS production, ATP deficiency, and impaired mitochondrial quality control (MQC) [90,91] (Table 1).

### 3.2. Parkinson’s Disease

PD is the second most common neurodegenerative disease in advanced age and the most frequent movement disorder [101]. PD arises upon degeneration of dopaminergic neurons of the substantia nigra pars compacta of the midbrain. Individuals with PD typically show rigidity, resting tremors, bradykinesia, and a hunched posture (camptocormia) [102]. PD can also be associated with non-motor signs and symptoms, including neurobehavioral disorders (e.g., depression, anxiety), cognitive impairment, and autonomic dysfunction [102]. Serum NF-L levels are higher in patients with PD than in healthy controls, increase during disease progression, and correlate with PD severity [96]. Moreover, circulating levels of NF-L support the differentiation of idiopathic PD from atypical Parkinsonian syndromes [103,104]. The discrimination power and accuracy of NF-L for distinguishing individuals with PD from those with other Parkinsonian disorders increase substantially when combined with amyloid β42 (Aβ42), phosphorylated tau (p-tau), total tau, and total α-synuclein (α-syn) in CSF [105]. High levels of NF-L in plasma/serum/CSF of PD patients have also been associated with a greater probability of developing mild cognitive impairments or dementia [106,107]. In two independent studies, Aamodt et al. [106] and Buhmann et al. [108] measured circulating NF-L levels in individuals with PD at different disease stages and found that NF-L levels varied according to the UPDRS III (Unified Parkinson’s Disease Rating Scale, Part III) score. No associations were identified with the Mattis Dementia Rating Scale (DRS-2) [106] or the Montreal Cognitive Assessment (MoCA) score [108]. Furthermore, results from the Biomarkers in Parkinson’s Disease (MARK-PD) study indicated that circulating NFL levels could predict the severity and progression of cognitive decline (DRS-2 change in plasma; decrease in MoCA score > 2 points in serum) but not motor aggravation (UPDRS III ≥ 5 points in plasma; increased UPDRS III > 4 points in serum) [106,108,109]. PD patients with serum NF-L levels over the age-adjusted 95th percentile showed a significantly more rapid cognitive decline than those with lower NF-L concentrations, suggesting that age influences NF-L variability [108]. Furthermore, a recent study found that baseline plasma NF-L levels were positively related to the development of psychotic symptoms in individuals with PD over up to seven years of follow-up [110]. Urso et al. [95] showed that NF-L levels in the CSF and especially in serum were associated with the worsening of non-motor symptoms; in particular, the progression of depression and anxiety. In an A53T-α-syn transgenic mouse model of PD, elevated NF-L levels in serum and CSF were positively correlated with the number and size of α-syn neuronal inclusions, a typical feature of PD pathology [93]. CSF α-syn acts as a mediator between NF-L and motor progression [92]. Plasma GFAP is another candidate biomarker of PD. Plasma levels of GFAP and NF-L are both related to motor dysfunction severity; however, NF-L seems to be a stronger predictor of PD progression [94]. The implementation of NF-L levels as a biomarker in clinical practice has long been awaited. Despite having low specificity for PD, NF-L has shown high sensitivity and is currently one of the best, easily accessible, blood-based PD biomarkers with diagnostic and prognostic values [92,96,97] (Table 1).

### 3.3. Charcot–Marie–Tooth

Charcot–Marie–Tooth (CMT) is an autosomal dominant (the most frequent), autosomal recessive, and X-linked disease existing in different clinical forms (i.e., demyelinating CMT1, axonal CMT2, and intermediate I-CMT). In CMT1, there is a lower motor nerve conduction velocity (MNCV) of the upper limb and a predominant alteration of Schwann cells that are fundamental for saltatory conduction [111]. CMT2 is characterized by a higher MNCV and is mainly caused by axonal degeneration [111]. Finally, in I-CMT, MNCV values are intermediate between those observed in CMT1 and CMT2 [111]. CMT appears to be an inherited peripheral neuropathy accompanied by a hereditary neuromuscular disorder. CMT patients show an indolent sensorimotor polyneuropathy that progresses very slowly in a length-dependent manner, pes cavus, hip dysplasia, tremor, restless legs syndrome, or hearing loss [55,112]. In this disease, NEFL and NEFH genes are often mutated, determining Nfl aggregation and altered phosphorylation, mainly of the head domain, and aberrant motility of neuronal mitochondria [55,113,114]. NEFL mutations can be either dominantly inherited missense mutations or recessively inherited nonsense mutations [115]. Therefore, both the presence of mutated NEFL protein and its absence can induce the disease [116]. In cultured cells, NEFL mutations affect their ability to generate Nfl aggregates and to stop Nfl assembly [117,118]. In terms of age of onset, mutations in rod and tail domains induce a later manifestation of the disease than those in the head domain of the NEFL, which are, therefore, responsible for more severe disease [119]. Mutations in the head and rod domains of NEFL are frequently found in CMT1 and CMT2 diseases [120,121]. Electron microscopy studies of sural nerve biopsies from CMT patients with missense NEFL mutations revealed atrophied axons with Nfl depletion and rare swellings filled with Nfl polymers [116]. In CMT patients with a nonsense mutation, axonal atrophy and loss were associated with the complete absence of Nfl [116]. These findings suggest that the organization or distribution of Nfls along the axons is involved in CMT [116]. NF-L is expressed in parallel with peripherin, with which it interacts during the formation of heteropolymers. Peripherin is involved in determining the morphology, maturation, and differentiation of neurons as well as in the regeneration of axons. Its assembly depends on the Ras-associated binding 7 (RAB7) protein, which is frequently mutated (RAB7K126R) in CMT2B, resulting in an altered interaction with peripherin and a lack of axonal regeneration [122]. Recent studies have demonstrated that the aberrant assembly of NF-L mutants is attributable to altered levels of O-linked betaN-acetylglucosamine (O-GlcNAc) [119]. Indeed, NF-L contains O-GlcNAc sites, which in mutants are not correctly O-GlcNAcetylated, which are fundamental for the formation of protein–protein interactions with itself and α-internexin [119].

Rossor et al. [99] evaluated the potential of plasma NF-L as a biomarker of CMT (Table 1). They found that plasma NF-L concentrations were higher in patients with CMT1B, CMT1X, and CMT2A, but not CMT2E, compared with controls [99]. These findings are consistent with a previous study in which NF-L expression was found to be reduced in cutaneous nerve fibers of patients with CMT2E [100]. However, plasma NF-L is weakly correlated with the clinical severity of CMT [98]. Indeed, a recent study found no correlation between changes in plasma NF-L levels over time and disease severity [123]. It is noteworthy that plasma levels of NF-L change over time with different trajectories depending on the disease subtype [99]. Altogether, these discordant data indicate that the role of NF-L in CMT deserves further investigation.

## 4. Intermediate Filaments in Skeletal Muscle Aging: The Role of Vimentin

Sarcomeres are the basic units producing force during muscle contraction. Force-generating capacity is an emerging attribute of sarcomeric proteins [124]. Alterations and/or mutations in single proteins, such as those composing the sarcomere thick and/or thin filaments, as well as those of the Z- and M-lines, are sufficient to affect sarcomere function. Sarcomere architecture relies mostly on the two cytoplasmatic IFs, vimentin and desmin [125]. Vimentin and desmin form a three-dimensional scaffold at the level of Z-disks. Desmin composes Z-disks of striated muscles and vimentin those of differentiating cells, including myoblasts [125]. Vimentin and desmin are both present during myogenesis as longitudinally organized and randomly distributed filaments, respectively [126]. Such arrangements likely provide a guide over which the cylindrical form of developing myotubes and parallel myofibers alignment occur [127]. Because newly synthesized desmin replaces pre-existing vimentin structures, desmin distribution in muscle fibers reflects that of vimentin. These filaments bear tension and preserve the structure and mechanical integrity of cells [128]. Vimentin and desmin also control mitochondrial bioenergetics and influence the mobility and anchoring of mitochondria within the cytoplasm [129].

Vimentin is organized in a structure encompassing five protofibrils per filament [130]. Of particular interest is the abundance of cysteine residues in the protein polymer that likely affects protein-folding in quaternary structures and generates cysteine codes [49]. Such an organization is compatible with cysteine residues in the proximity of each protofibril, where they are critical for filament assembly and cytoskeletal networks in human vimentin. Indeed, vimentin mutations, while having little or no influence on filament morphology, affect filament networking [131,132]. In this regard, cysteine clustering may amplify the impact of cysteine modifications on the organization of the filament and the network. In further support of the relevance of such a role is the finding of cysteine residues with similar functions in GFAP and desmin [132,133]. Notably, vimentin copolymers with desmin or GFAP show varying levels of cysteine-crosslinked dimers ratios, likely indicating that polymer co-assembly also affects the molecular organization of the filament [134,135].

A set of cysteine modifications have been related to filament disruption and/or remodeling. For instance, glutathionylation blunts filament elongation [136], while disulfide crosslinking triggers their aggregation and impairs their assembly [137,138]. Incubation with 4-hydroxynonenal allows shorter and wider filament formations, while filament bundling occurs with electrophilic compounds [137]. Of all these, glutathionylation can induce filament severing [136], thereby indicating that the proportion of modified cysteine residues is more relevant to filament disruption and/or remodeling. Vimentin dimers formed by disulfide bonds and/or chemically cysteine crosslinking have also been identified [131,134,135,138].

Redox proteomics analysis of muscle biopsies revealed age-related oxidized cysteine signatures in sarcomere proteins and reduced physical performance (walking speed), muscle function (muscle strength and power), and cardiopulmonary fitness (VO_2_ max) [139]. These results add to previous findings showing an association between cysteine oxidation in sarcomere proteins and the structure and function of contractile units. For instance, competitive glutathionylation and nitrosylation of fast-twitch troponin I cysteine 134 modulate calcium sensitivity of muscle fibers and muscle force [140]. Oxidative modifications have also been identified in the large protein titin, with negative consequences on elasticity and stiffness [141].

While these findings provide a solid ground for designing larger and more focused studies, the characterization of reactive cysteine residues in IFs remains challenging [142]. Methods involving cysteine reactive probes combined with biochemical approaches, labeled electrophilic lipids and glutathione, and antibodies against cysteine modifications have been implemented in vitro and in vivo [143]. However, the gold standard for the assessment of cysteine’s post-translational modifications is mass spectrometry [143,144,145], which should be applied at both cellular and subcellular levels. This technical constraint hampers the translational application of such a measure and limits the understanding of these modifications at the clinical level. Indeed, most studies investigating the role of cysteine modifications in filament assembly have been performed in vitro [136,137,138]. Cysteine mutants have been mostly used for understanding the functional relevance of cysteine residues in cellular protein oxidation and lipoxidation [146,147,148]. Experimental models of vimentin-deficient cells are less competent in integrating stress responses as they neither adequately position organelles in the cell nor efficiently recruit ubiquitinated proteins [131]. Finally, several cellular factors influence cysteine reactivity and/or accessibility and pose additional challenges for conducting such studies.

## 5. The Cytoskeletal Regulation of Mitochondria

Mitochondria are best known for their role in energy provision in the form of ATP; however, these organelles serve a plethora of different functions and are the main executors of cell death, either apoptotic or necrotic [149]. Mitochondria rely upon several mechanisms to maintain their homeostasis and avoid cell death, including mitochondrial biogenesis, dynamics, and mitophagy, altogether referred to as MQC processes [149]. During fission, mitochondria are portioned into two distinct entities that separate the healthy/partially damaged components from the totally damaged portions that are directed toward mitophagy. Partially deficient and/or dysfunctional mitochondria undergo fusion by merging their membranes with those of new mitochondria obtained through mitochondrial biogenesis, thus reacquiring normal physiological activities [150,151]. The net balance of fusion–fission processes while determining the shape of the organelle also defines the entire cellular network, whose architecture results from mitochondria-to-mitochondria contacts and, from these, with neighboring organelles and the cytoskeleton [152,153]. When mitochondrial relocation is needed, mitochondria detach from the network and become mitochondrial “units” that are easier to transport. Movement and morphological changes are tightly related to the cytoskeleton, which intervenes in regulating these processes [152]. Mitochondria interact with and modulate all three main components of the cytoskeleton (i.e., actin microfilaments, microtubules, and IFs). The contact and modulation of IFs have been actively investigated, especially in the context of neurodegenerative conditions. However, the interrelationship between mitochondrial function and microtubules and actin microfilaments is not less relevant.

**Figure 2:**
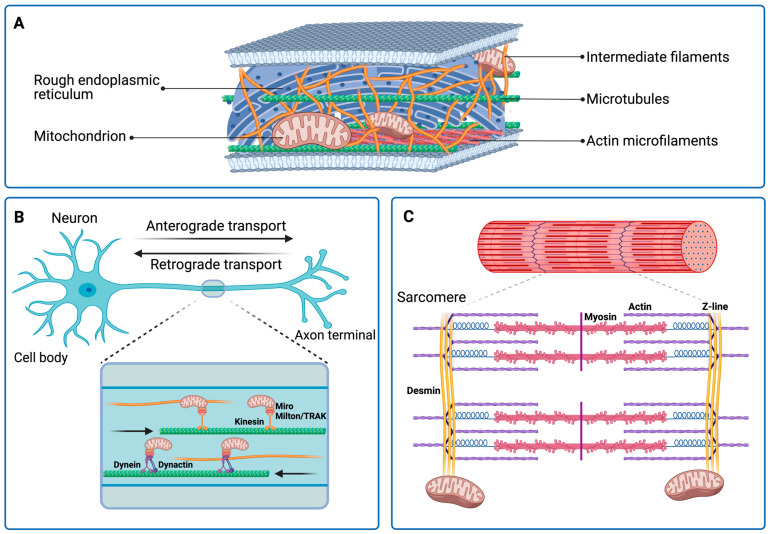
Schematic representation of the cytoskeletal organization (**A**) and cytoskeletal–mitochondria interactions in a neuron (**B**) and the sarcomere (**C**). Created in https://BioRender.com.

Mitochondria move along the cytoskeleton, mainly along microtubules, to which they are anchored by the motor proteins dynein and kinesin. Microtubules are composed of heterodimers of α- and β-tubulin that form cylindrical structures of about 25 nm in diameter and are involved in maintaining cell and organelles shape, size, trafficking and anchoring, and chromosome segregation during cell division [128,129]. Microfilaments are made of two intertwined filaments of G-actin to form F-actin with a diameter of about 7 nm. Due to their ability to polymerize/depolymerize depending on the stimuli, microtubules can actively modify the shape of the cell and induce its motility [128]. In undifferentiated cells, microtubules are arranged radially, with their minus ends facing the center of the cell and the plus ends facing outwards. Mitochondria motility toward the minus end of microtubules is driven by dynein with the support of its activator dynactin, while motility toward the plus end is mediated by kinesin-1. In neurons, dynein is responsible for mitochondria movement from the axon to the cell body (retrograde transport), whereas kinesin allows transportation from the cell body to the axon (anterograde transport) [154,155]. Dynein and kinesin-1 use adaptor complexes, such as the Ca^2+^-binding GTPase Miro and Milton/trafficking kinesin protein (TRAK), to bind to mitochondria (Figure 2).

In Drosophila, Milton interacts with mitochondria via Miro, located at the mitochondrial outer membrane. Miro mutants exhibit a loss of mitochondria in axons and impaired anterograde traffic, which is lethal. In mice, the neuronal-specific deletion of Miro1 causes both neurodevelopmental and neurodegenerative defects [29,30,156,157]. The cytoskeleton plays a crucial role in the transportation of molecules and organelles, especially mitochondria, in nerve cells. Aberrant cytoskeletal variations can trigger synaptic dysfunction, progressive neuronal loss, abnormal energy metabolism, and mitochondrial alterations and diseases [158,159].

Derangements of motor adapter proteins also induce alterations in mitochondrial transportation in dendrites and axons, resulting in the blockade of synaptic transmissions and mitochondrial dynamics found in ALS, PD, and CMT [160]. The anterograde movement of mitochondria with axons is mediated by the kinesin family member 5A (KIF5A) through its interaction with the adapters TRAK1 and Miro1/2. Mutations in the gene coding for KIF5A impact the rate of axonal anterograde transportation of mitochondria and Nfl proteins. The mutation of KIF5A in zebrafish decreases the number of mitochondria and the speed at which they move along the axon [161]. KIF5A-knockout mice accumulate Nfls in the soma of peripheral sensory neurons, resulting in axonal reductions, loss of large axons, and degeneration [162]. These mutations have been found in ALS and, in rare cases, CMT2 [163,164,165,166]. Mutations in the dynein 1 heavy chain 1 (DYNC1H1) gene impair retrograde mitochondrial transportation, leading to increased death of cultured dorsal root ganglion neurons [167], sensory neuropathy, and loss of motor function in mice [168]. These mutations have been identified in individuals with CMT [169]. In ALS patients, the typical swelling of the AIS in motor neurons, rich in lysosomes, mitochondria, vesicles, and IFs, is an indicator of defects in cytoskeletal and axonal transport [170]. Moreover, in ALS, mutations and rare cargo variants in α-tubulin 4a, encoded by the TUBA4A (Tubulin Alpha 4a) gene, have been identified that dimerize and fail to be efficiently incorporated into microtubules in vitro, thus destabilizing the microtubule network and limiting its polymerization [170]. In PD, mutations in PTEN-induced putative kinase 1 (PINK1), E3 ubiquitin ligase (Parkin), or leucine-rich repeat kinase 2 (LRRK2) blunt the elimination of damaged mitochondria, thereby indicating that the PINK1- and Parkin-dependent degradation of Miro is necessary to immobilize damaged mitochondria and address them toward mitophagy [171]. A dysregulation of PINK1 and Parkin has also been identified in individuals with sporadic ALS [172]. Parkin levels can be reduced following the overexpression of TARDBP, which alters proteasomal activity, causing the accumulation of PINK1, which, in turn, is responsible for mitochondrial dysfunction [173]. Similarly, ALS-related FUS (FUS RNA Binding Protein) mutants exhibit an aggregation of PINK1 and Parkin proteins and ubiquitination of Miro1, resulting in the blockade of axonal transportation of mitochondria [174]. Miro is essential for the axonal retrograde trafficking of mitochondria, but it also intervenes in the anterograde traffic of axons. In this latter case, the protein factor RIC-7 seems to be required, and its localization is regulated by the cooperation of Miro/MIRO-1 and metaxin2/MTX-2 [175]. The conformation of tubulin dimers within the microtubule lattice also plays a role in regulating mitochondrial transport. A recent study has shown that mitochondria moving within the microtubule bundle are hindered by the increased straightness of elongated tubulin-enriched microtubules, while mitochondria moving to the edge of the microtubule bundle are transported at higher speeds because the molecular conformation of tubulin influences kinesin-binding and the processing speed in axons [176]. A greater density of elongated dimers makes the microtubules straighter, thus favoring a more efficient movement of kinesin, which results in an increased speed of transport [176].

Actin microfilaments are also key to cell division, fission, mitophagy, and mitochondrial morphogenesis. When mitochondria are damaged, short actin filaments, created by cooperation with the inverted formin 2 (INF2) protein and the mitochondria-located Spire1C protein, polymerize and span mitochondria–endoplasmic reticulum (ER) contacts [177]. This promotes the wrapping of ER tubules around the mitochondria [178]. Such a pre-constriction reduces the cross-sectional diameter of the organelle and facilitates a ring-shaped assembly and hydrolysis-mediated constriction of dynamin-related protein-1 (DRP1) oligomers on the outer mitochondrial membrane. Herein, also the mitochondrial fission protein 1 (FIS1) is localized [179], thus initiating the fission process. Through a similar mechanism, actin filaments equally distribute healthy and damaged mitochondria of the mother cell to the daughter cells during cell division [129]. A mutation in the INF2 gene has been implicated in CMT1E [160]. Furthermore, FIS1 interacts with the ALS-associated gene C9orf72 [180], and mutations in FIS1 have been shown to prevent the proper disposal of aberrant mitochondria [181]. Finally, myosin, an actin-based molecular motor, in its myosin XIX isoform, is a plus-end directed motor protein located at the outer mitochondrial membrane, where it directs mitochondrial movement [182,183,184]. Its localization depends on Miro proteins, which, therefore, intervene not only in microtubule-based mitochondrial motility but also in actin-based motility [185]. Finally, in MNs of Drosophila, myosins V and VI anchor mitochondria to actin filaments and oppose microtubule-based motility. Mutations in myosin IX are associated with CMT2 neuropathies [186].

## 6. Conclusions

While largely recognized as cell scaffolds, cytoskeletal proteins, and especially IFs, have shown additional and, in some instances, more relevant functional roles with key implications in physiological and stress signaling. Their networking ability and interaction with cellular organelles that influence function, positioning, and oxidative species regulation have placed these polymers in the spotlight as crucial platforms for protein and organelle modulators. Additional research is needed to dissect these functions. However, from a translational point of view, harnessing the properties of IFs in physiological conditions and under stress may help unveil the mechanisms of chronic disease and eventually develop therapeutic interventions. Such interventions may include either drugs or mechanical stimulations and dietary interventions to restore and/or enhance cytoskeletal integrity. The treatment of choice would depend on the specific disease and the cytoskeletal components that are targeted.

## Figures and Tables

**Table 1:** List of the main studies describing the implication of neurofilaments in neurodegeneration.

Condition	Main Pathological Trait	Circulating Marker(s)	Reference(s)
ALS	Motor neuron disorder	↑ NF-L, p-NF-H, anti-NF-L	[87,89]
PD	Death of dopaminergic neurons in the substantia nigra pars compacta	↑ GFAP, α-synuclein, NF-L	[92,93,94,95,96,97]
CMT1B	Demyelinating peripheral neuropathy	↑ NF-L	[98,99]
CMT1X	Demyelinating peripheral neuropathy	↑ NF-L	[98,99]
CMT2A	Axonal peripheral neuropathy	↑ NF-L	[98,99]
CMT2E	Axonal peripheral neuropathy	↓ NF-L	[100]

Abbreviations: ALS, amyotrophic lateral sclerosis; CMT, Charcot–Marie–Tooth; GFAP, glial fibrillary acidic protein; NF-L, low molecular weight neurofilament; PD, Parkinson’s disease; p-NF-H, phosphorylated high molecular weight neurofilament protein.

## References

[1] Pegoraro A.F., Janmey P., Weitz D.A. (2017). Mechanical properties of the cytoskeleton and cells. Cold Spring Harb. Perspect. Biol..

[2] Phillip J.M., Wu P.H., Gilkes D.M., Williams W., McGovern S., Daya J., Chen J., Aifuwa I., Lee J.S.H., Fan R. (2017). Biophysical and biomolecular determination of cellular age in humans. Nat. Biomed. Eng..

[3] Seawright J.W., Sreenivasappa H., Gibbs H.C., Padgham S., Shin S.Y., Chaponnier C., Yeh A.T., Trzeciakowski J.P., Woodman C.R., Trache A. (2018). Vascular smooth muscle contractile function declines with age in skeletal muscle feed arteries. Front. Physiol..

[4] Zhu Y., Qiu H., Trzeciakowski J.P., Sun Z., Li Z., Hong Z., Hill M.A., Hunter W.C., Vatner D.E., Vatner S.F. (2012). Temporal analysis of vascular smooth muscle cell elasticity and adhesion reveals oscillation waveforms that differ with aging. Aging Cell.

[5] del Campo L., Sánchez-López A., Salaices M., von Kleeck R.A., Expósito E., González-Gómez C., Cussó L., Guzmán-Martínez G., Ruiz-Cabello J., Desco M. (2019). Vascular smooth muscle cell-specific progerin expression in a mouse model of Hutchinson-Gilford progeria syndrome promotes arterial stiffness: Therapeutic effect of dietary nitrite. Aging Cell.

[6] Qiu H., Zhu Y., Sun Z., Trzeciakowski J.P., Gansner M., Depre C., Resuello R.R.G., Natividad F.F., Hunter W.C., Genin G.M. (2010). Short communication: Vascular smooth muscle cell stiffness as a mechanism for increased aortic stiffness with aging. Circ. Res..

[7] Herum K.M., Choppe J., Kumar A., Engler A.J., McCulloch A.D. (2017). Mechanical regulation of cardiac fibroblast profibrotic phenotypes. Mol. Biol. Cell.

[8] Picca A., Calvani R., Manes-Gravina E., Spaziani L., Landi F., Bernabei R., Marzetti E. (2017). Bone-muscle crosstalk: Unraveling new therapeutic targets for osteoporosis. Curr. Pharm. Des..

[9] Ferrucci L., Baroni M., Ranchelli A., Lauretani F., Maggio M., Mecocci P., Ruggiero C. (2014). Interaction between bone and muscle in older persons with mobility limitations. Curr. Pharm. Des..

[10] Marzetti E., Lozanoska-Ochser B., Calvani R., Landi F., Coelho-Júnior H.J., Picca A. (2024). Restoring mitochondrial function and muscle satellite cell signaling: Remedies against age-related sarcopenia. Biomolecules.

[11] Arosio B., Picca A. (2024). The biological roots of the sex-frailty paradox. Exp. Gerontol..

[12] Tiede-Lewis L.A.M., Xie Y., Hulbert M.A., Campos R., Dallas M.R., Dusevich V., Bonewald L.F., Dallas S.L. (2017). Degeneration of the osteocyte network in the C57BL/6 mouse model of aging. Aging.

[13] Tiede-Lewis L.A.M., Dallas S.L. (2019). Changes in the osteocyte lacunocanalicular network with aging. Bone.

[14] Glatt V., Canalis E., Stadmeyer L., Bouxsein M.L. (2007). Age-related changes in trabecular architecture differ in female and male C57BL/6J mice. J. Bone Miner. Res..

[15] Morrell A.E., Robinson S.T., Silva M.J., Guo X.E. (2020). Mechanosensitive Ca^2+^ signaling and coordination is diminished in osteocytes of aged mice during ex vivo tibial loading. Connect. Tissue Res..

[16] González-Bermúdez B., Kobayashi H., Abarca-Ortega A., Córcoles-Lucas M., González-Sánchez M., De la Fuente M., Guinea G.V., Elices M., Plaza G.R. (2022). Aging is accompanied by T-cell Stiffening and reduced interstitial migration through dysfunctional nuclear organization. Immunology.

[17] Racine M.L., Dinenno F.A. (2019). Reduced deformability contributes to impaired deoxygenation-induced ATP release from red blood cells of older adult humans. J. Physiol..

[18] Lawrence E.J., Boucher E., Mandato C.A. (2016). Mitochondria-cytoskeleton associations in mammalian cytokinesis. Cell Div..

[19] Knowles M.K., Guenza M.G., Capaldi R.A., Marcus A.H. (2002). Cytoskeletal-assisted dynamics of the mitochondrial reticulum in living cells. Proc. Natl. Acad. Sci. USA.

[20] Kuznetsov A.V., Javadov S., Grimm M., Margreiter R., Ausserlechner M.J., Hagenbuchner J. (2020). Crosstalk between mitochondria and cytoskeleton in cardiac cells. Cells.

[21] Sies H., Jones D.P. (2020). Reactive oxygen species (ROS) as pleiotropic physiological signalling agents. Nat. Rev. Mol. Cell Biol..

[22] Varesi A., Chirumbolo S., Campagnoli L.I.M., Pierella E., Piccini G.B., Carrara A., Ricevuti G., Scassellati C., Bonvicini C., Pascale A. (2022). The role of antioxidants in the interplay between oxidative stress and senescence. Antioxidants.

[23] Di Lorenzo R., Chimienti G., Picca A., Trisolini L., Latronico T., Liuzzi G.M., Pesce V., Leeuwenburgh C., Lezza A.M.S. (2024). Resveratrol impinges on retrograde communication without inducing mitochondrial biogenesis in aged rat soleus muscle. Exp. Gerontol..

[24] Guéraud F., Atalay M., Bresgen N., Cipak A., Eckl P.M., Huc L., Jouanin I., Siems W., Uchida K. (2010). Chemistry and biochemistry of lipid peroxidation products. Free Radic. Res..

[25] Viedma-Poyatos Á., González-Jiménez P., Langlois O., Company-Marín I., Spickett C.M., Pérez-Sala D. (2021). Protein lipoxidation: Basic concepts and emerging roles. Antioxidants.

[26] Perrot R., Berges R., Bocquet A., Eyer J. (2008). Review of the multiple aspects of neurofilament functions, and their possible contribution to neurodegeneration. Mol. Neurobiol..

[27] Didonna A., Opal P. (2019). The role of neurofilament aggregation in neurodegeneration: Lessons from rare inherited neurological disorders. Mol. Neurodegener..

[28] Verde F., Otto M., Silani V. (2021). neurofilament light chain as biomarker for amyotrophic lateral sclerosis and frontotemporal dementia. Front. Neurosci..

[29] van Spronsen M., Mikhaylova M., Lipka J., Schlager M.A., van den Heuvel D.J., Kuijpers M., Wulf P.S., Keijzer N., Demmers J., Kapitein L.C. (2013). TRAK/Milton motor-adaptor proteins steer mitochondrial trafficking to axons and dendrites. Neuron.

[30] López-Doménech G., Higgs N.F., Vaccaro V., Roš H., Arancibia-Cárcamo I.L., MacAskill A.F., Kittler J.T. (2016). Loss of dendritic complexity precedes neurodegeneration in a mouse model with disrupted mitochondrial distribution in mature dendrites. Cell Rep..

[31] Sanghvi-Shah R., Weber G.F. (2017). Intermediate filaments at the junction of mechanotransduction, migration, and development. Front. Cell Dev. Biol..

[32] Schweizer J., Bowden P.E., Coulombe P.A., Langbein L., Lane E.B., Magin T.M., Maltais L., Omary M.B., Parry D.A.D., Rogers M.A. (2006). New consensus nomenclature for mammalian keratins. J. Cell Biol..

[33] Dutour-Provenzano G., Etienne-Manneville S. (2021). Intermediate filaments. Curr. Biol..

[34] Toivola D.M., Tao G.Z., Habtezion A., Liao J., Omary M.B. (2005). Cellular integrity plus: Organelle-related and protein-targeting functions of intermediate filaments. Trends Cell Biol..

[35] Iwatsuki H., Suda M. (2010). Seven kinds of intermediate filament networks in the cytoplasm of polarized cells: Structure and function. Acta Histochem. Cytochem..

[36] Lépinoux-Chambaud C., Eyer J. (2013). Review on intermediate filaments of the nervous system and their pathological alterations. Histochem. Cell Biol..

[37] Lund L.M., Kerr J.P., Lupinetti J., Zhang Y., Russell M.A., Bloch R.J., Bond M. (2012). Synemin isoforms differentially organize cell junctions and desmin filaments in neonatal cardiomyocytes. FASEB J..

[38] Michalczyk K., Ziman M. (2005). Nestin structure and predicted function in cellular cytoskeletal organisation. Histol. Histopathol..

[39] Duarte S., Viedma-Poyatos Á., Navarro-Carrasco E., Martínez A.E., Pajares M.A., Pérez-Sala D. (2019). Vimentin filaments interact with the actin cortex in mitosis allowing normal cell division. Nat. Commun..

[40] Jokhadar Š.Z., Stojković B., Vidak M., Sorčan T., Liovic M., Gouveia M., Travasso R.D.M., Derganc J. (2020). Cortical stiffness of keratinocytes measured by lateral indentation with optical tweezers. PLoS ONE.

[41] Ndiaye A.B., Koenderink G.H., Shemesh M. (2022). Intermediate filaments in cellular mechanoresponsiveness: Mediating cytoskeletal crosstalk from membrane to nucleus and back. Front. Cell Dev. Biol..

[42] Stenvall C.G.A., Nyström J.H., Butler-Hallissey C., Jansson T., Heikkilä T.R.H., Adam S.A., Foisner R., Goldman R.D., Ridge K.M., Toivola D.M. (2022). Cytoplasmic keratins couple with and maintain nuclear envelope integrity in colonic epithelial cells. Mol. Biol. Cell.

[43] Gross A., Zhou B., Bewersdorf L., Schwarz N., Schacht G.M., Boor P., Hoeft K., Hoffmann B., Fuchs E., Kramann R. (2022). Desmoplakin maintains transcellular keratin scaffolding and protects from intestinal injury. Cell. Mol. Gastroenterol. Hepatol..

[44] Fuchs E., Weber K. (1994). Intermediate filaments: Structure, dynamics, function, and disease. Annu. Rev. Biochem..

[45] Schreiber K.H., Kennedy B.K. (2013). When lamins go bad: Nuclear structure and disease. Cell.

[46] Graziano S., Coll-Bonfill N., Teodoro-Castro B., Kuppa S., Jackson J., Shashkova E., Mahajan U., Vindigni A., Antony E., Gonzalo S. (2021). Lamin A/C recruits ssdna protective proteins RPA and RAD51 to stalled replication forks to maintain fork stability. J. Biol. Chem..

[47] Georgatos S.D., Gounari F., Remington S. (1994). The beaded intermediate filaments and their potential functions in eye lens. Bioessays.

[48] Song S., Landsbury A., Dahm R., Liu Y., Zhang Q., Quinlan R.A. (2009). Functions of the intermediate filament cytoskeleton in the eye lens. J. Clin. Investig..

[49] Viedma-Poyatos Á., Pajares M.A., Pérez-Sala D. (2020). Type III intermediate filaments as targets and effectors of electrophiles and oxidants. Redox Biol..

[50] Yuan A., Rao M.V., Sasaki T., Chen Y., Kumar A., Veeranna, Liem R.K.H., Eyer J., Peterson A.C., Julien J.P. (2006). Alpha-internexin is structurally and functionally associated with the neurofilament triplet proteins in the mature CNS. J. Neurosci..

[51] Liem R.K.H., Messing A. (2009). Dysfunctions of neuronal and glial intermediate filaments in disease. J. Clin. Investig..

[52] Kirkcaldie M.T.K., Dwyer S.T. (2017). The third wave: Intermediate filaments in the maturing nervous system. Mol. Cell. Neurosci..

[53] Bomont P. (2021). The dazzling rise of neurofilaments: Physiological functions and roles as biomarkers. Curr. Opin. Cell Biol..

[54] Gentil B.J., Tibshirani M., Durham H.D. (2015). Neurofilament dynamics and involvement in neurological disorders. Cell Tissue Res..

[55] Kotaich F., Caillol D., Bomont P. (2023). Neurofilaments in health and Charcot-Marie-Tooth disease. Front. Cell Dev. Biol..

[56] Yuan A., Rao M.V., Veeranna, Nixon R.A. (2017). Neurofilaments and neurofilament proteins in health and disease. Cold Spring Harb. Perspect. Biol..

[57] Bomont P. (2016). Degradation of the intermediate filament family by gigaxonin. Methods Enzymol..

[58] Fauré J., Lachenal G., Court M., Hirrlinger J., Chatellard-Causse C., Blot B., Grange J., Schoehn G., Goldberg Y., Boyer V. (2006). Exosomes are released by cultured cortical neurones. Mol. Cell. Neurosci..

[59] Lachenal G., Pernet-Gallay K., Chivet M., Hemming F.J., Belly A., Bodon G., Blot B., Haase G., Goldberg Y., Sadoul R. (2011). Release of exosomes from differentiated neurons and its regulation by synaptic glutamatergic activity. Mol. Cell. Neurosci..

[60] Carare R.O., Bernardes-Silva M., Newman T.A., Page A.M., Nicoll J.A.R., Perry V.H., Weller R.O. (2008). Solutes, but not cells, drain from the brain parenchyma along basement membranes of capillaries and arteries: Significance for cerebral amyloid angiopathy and neuroimmunology. Neuropathol. Appl. Neurobiol..

[61] Khalil M., Teunissen C.E., Lehmann S., Otto M., Piehl F., Ziemssen T., Bittner S., Sormani M.P., Gattringer T., Abu-Rumeileh S. (2024). Neurofilaments as biomarkers in neurological disorders—Towards clinical application. Nat. Rev. Neurol..

[62] Kuhle J., Barro C., Andreasson U., Derfuss T., Lindberg R., Sandelius Å., Liman V., Norgren N., Blennow K., Zetterberg H. (2016). Comparison of three analytical platforms for quantification of the neurofilament light chain in blood samples: ELISA, electrochemiluminescence immunoassay and Simoa. Clin. Chem. Lab. Med..

[63] Truffi M., Garofalo M., Ricciardi A., Cotta Ramusino M., Perini G., Scaranzin S., Gastaldi M., Albasini S., Costa A., Chiavetta V. (2023). Neurofilament-light chain quantification by Simoa and Ella in plasma from patients with dementia: A comparative study. Sci. Rep..

[64] Meeker K.L., Butt O.H., Gordon B.A., Fagan A.M., Schindler S.E., Morris J.C., Benzinger T.L.S., Ances B.M. (2022). Cerebrospinal fluid neurofilament light chain is a marker of aging and white matter damage. Neurobiol. Dis..

[65] Schultz S.A., Strain J.F., Adedokun A., Wang Q., Preische O., Kuhle J., Flores S., Keefe S., Dincer A., Ances B.M. (2020). Serum neurofilament light chain levels are associated with white matter integrity in autosomal dominant Alzheimer’s disease. Neurobiol. Dis..

[66] Jung Y., Damoiseaux J.S. (2024). The potential of blood neurofilament light as a marker of neurodegeneration for Alzheimer’s disease. Brain.

[67] Longinetti E., Fang F. (2019). Epidemiology of amyotrophic lateral sclerosis: An update of recent literature. Curr. Opin. Neurol..

[68] Goutman S.A., Hardiman O., Al-Chalabi A., Chió A., Savelieff M.G., Kiernan M.C., Feldman E.L. (2022). Recent advances in the diagnosis and prognosis of amyotrophic lateral sclerosis. Lancet Neurol..

[69] Tosolini A.P., Sleigh J.N., Surana S., Rhymes E.R., Cahalan S.D., Schiavo G. (2022). BDNF-dependent modulation of axonal transport is selectively impaired in ALS. Acta Neuropathol. Commun..

[70] Štetkárová I., Ehler E. (2021). Diagnostics of amyotrophic lateral sclerosis: Up to date. Diagnostics.

[71] Sleigh J.N., Tosolini A.P., Gordon D., Devoy A., Fratta P., Fisher E.M.C., Talbot K., Schiavo G. (2020). Mice carrying ALS mutant TDP-43, but not mutant FUS, display in vivo defects in axonal transport of signaling endosomes. Cell Rep..

[72] Millecamps S., Julien J.P. (2013). Axonal transport deficits and neurodegenerative diseases. Nat. Rev. Neurosci..

[73] Lefebvre-Omar C., Liu E., Dalle C., d’Incamps B.L., Bigou S., Daube C., Karpf L., Davenne M., Robil N., Jost Mousseau C. (2023). Neurofilament accumulations in amyotrophic lateral sclerosis patients’ motor neurons impair axonal initial segment integrity. Cell. Mol. Life Sci..

[74] Garcia M.L., Singleton A.B., Hernandez D., Ward C.M., Evey C., Sapp P.A., Hardy J., Brown R.H., Cleveland D.W. (2006). Mutations in neurofilament genes are not a significant primary cause of non-SOD1-mediated amyotrophic lateral sclerosis. Neurobiol. Dis..

[75] Al-Chalabi A., Miller C.C.J. (2003). Neurofilaments and neurological disease. Bioessays.

[76] Figlewicz D.A., Rouleau G.A., Krizus A., Julien J.P. (1993). Polymorphism in the multi-phosphorylation domain of the human neurofilament heavy-subunit-encoding gene. Gene.

[77] Al-Chalabi A., Andersen P.M., Nilsson P., Chioza B., Andersson J.L., Russ C., Shaw C.E., Powell J.F., Leigh P.N. (1999). Deletions of the heavy neurofilament subunit tail in amyotrophic lateral sclerosis. Hum. Mol. Genet..

[78] Figlewicz D.A., Krizus A., Martinoli M.G., Meininger V., Dib M., Rouleau G.A., Julien J.P. (1994). Variants of the heavy neurofilament subunit are associated with the development of amyotrophic lateral sclerosis. Hum. Mol. Genet..

[79] Tomkins J., Usher P., Slade J.Y., Ince P.G., Curtis A., Bushby K., Shaw P.J. (1998). Novel insertion in the KSP region of the neurofilament heavy gene in amyotrophic lateral sclerosis (ALS). Neuroreport.

[80] Mukai H., Toshimori M., Shibata H., Kitagawa M., Shimakawa M., Miyahara M., Sunakawa H., Ono Y. (1996). PKN associates and phosphorylates the head-rod domain of neurofilament protein. J. Biol. Chem..

[81] Manser C., Stevenson A., Banner S., Davies J., Tudor E.L., Ono Y., Nigel Leigh P., McLoughlin D.M., Shaw C.E., Miller C.C.J. (2008). Deregulation of PKN1 activity disrupts neurofilament organisation and axonal transport. FEBS Lett..

[82] Shea T.B., Chan W.K.H. (2008). Regulation of neurofilament dynamics by phosphorylation. Eur. J. Neurosci..

[83] Ackerley S., Grierson A.J., Banner S., Perkinton M.S., Brownlees J., Byers H.L., Ward M., Thornhill P., Hussain K., Waby J.S. (2004). P38α stress-activated protein kinase phosphorylates neurofilaments and is associated with neurofilament pathology in amyotrophic lateral sclerosis. Mol. Cell. Neurosci..

[84] Chen H., Qian K., Du Z., Cao J., Petersen A., Liu H., Blackbourn L.W., Huang C.L., Errigo A., Yin Y. (2014). Modeling ALS with iPSCs reveals that mutant SOD1 misregulates neurofilament balance in motor neurons. Cell Stem Cell.

[85] McMackin R., Bede P., Ingre C., Malaspina A., Hardiman O. (2023). Biomarkers in amyotrophic lateral sclerosis: Current status and future prospects. Nat. Rev. Neurol..

[86] Steinacker P., Huss A., Mayer B., Grehl T., Grosskreutz J., Borck G., Kuhle J., Lulé D., Meyer T., Oeckl P. (2017). Diagnostic and prognostic significance of neurofilament light chain NF-L, but not progranulin and S100B, in the course of amyotrophic lateral sclerosis: Data from the German MND-Net. Amyotroph. Lateral Scler. Front. Degener..

[87] Rossi D., Volanti P., Brambilla L., Colletti T., Spataro R., La Bella V. (2018). CSF neurofilament proteins as diagnostic and prognostic biomarkers for amyotrophic lateral sclerosis. J. Neurol..

[88] Meyer T., Schumann P., Weydt P., Petri S., Koc Y., Spittel S., Bernsen S., Günther R., Weishaupt J.H., Dreger M. (2023). Neurofilament light-chain response during therapy with antisense oligonucleotide tofersen in SOD1-related ALS: Treatment experience in clinical practice. Muscle Nerve.

[89] Puentes F., Topping J., Kuhle J., Van Der Star B.J., Douiri A., Giovannoni G., Baker D., Amor S., Malaspina A. (2014). Immune reactivity to neurofilament proteins in the clinical staging of amyotrophic lateral sclerosis. J. Neurol. Neurosurg. Psychiatry.

[90] Singh T., Jiao Y., Ferrando L.M., Yablonska S., Li F., Horoszko E.C., Lacomis D., Friedlander R.M., Carlisle D.L. (2021). Neuronal mitochondrial dysfunction in sporadic amyotrophic lateral sclerosis is developmentally regulated. Sci. Rep..

[91] Calió M.L., Henriques E., Siena A., Bertoncini C.R.A., Gil-Mohapel J., Rosenstock T.R. (2020). Mitochondrial dysfunction, neurogenesis, and epigenetics: Putative implications for amyotrophic lateral sclerosis neurodegeneration and treatment. Front. Neurosci..

[92] Liu Y., Dou K., Xue L., Li X., Xie A. (2022). Neurofilament light as a biomarker for motor decline in Parkinson’s disease. Front. Neurosci..

[93] Bacioglu M., Maia L.F., Preische O., Schelle J., Apel A., Kaeser S.A., Schweighauser M., Eninger T., Lambert M., Pilotto A. (2016). Neurofilament light chain in blood and CSF as marker of disease progression in mouse models and in neurodegenerative diseases. Neuron.

[94] Pilotto A., Ashton N.J., Lupini A., Battaglio B., Zatti C., Trasciatti C., Gipponi S., Cottini E., Grossi I., Salvi A. (2024). Plasma NfL, GFAP, amyloid, and p-Tau species as prognostic biomarkers in Parkinson’s disease. J. Neurol..

[95] Urso D., Batzu L., Logroscino G., Ray Chaudhuri K., Pereira J.B. (2023). Neurofilament light predicts worse nonmotor symptoms and depression in Parkinson’s disease. Neurobiol. Dis..

[96] Mollenhauer B., Dakna M., Kruse N., Galasko D., Foroud T., Zetterberg H., Schade S., Gera R.G., Wang W., Gao F. (2020). Validation of serum neurofilament light chain as a biomarker of Parkinson’s disease progression. Mov. Disord..

[97] Halloway S., Desai P., Beck T., Aggarwal N., Agarwal P., Evans D., Rajan K.B. (2022). Association of neurofilament light with the development and severity of Parkinson disease. Neurology.

[98] Millere E., Rots D., Simrén J., Ashton N.J., Kupats E., Micule I., Priedite V., Kurjane N., Blennow K., Gailite L. (2021). Plasma neurofilament light chain as a potential biomarker in Charcot-Marie-Tooth disease. Eur. J. Neurol..

[99] Rossor A.M., Kapoor M., Wellington H., Spaulding E., Sleigh J.N., Burgess R.W., Laura M., Zetterberg H., Bacha A., Wu X. (2022). A Longitudinal and cross-sectional study of plasma neurofilament light chain concentration in Charcot-Marie-Tooth disease. J. Peripher. Nerv. Syst..

[100] Pisciotta C., Bai Y., Brennan K.M., Wu X., Grider T., Feely S., Wang S., Moore S., Siskind C., Gonzalez M. (2015). Reduced neurofilament expression in cutaneous nerve fibers of patients with CMT2E. Neurology.

[101] Ben-Shlomo Y., Darweesh S., Llibre-Guerra J., Marras C., San Luciano M., Tanner C. (2024). The epidemiology of Parkinson’s disease. Lancet.

[102] Beitz J.M. (2014). Parkinson’s disease: A review. Front. Biosci. (Schol. Ed.).

[103] Hansson O., Janelidze S., Hall S., Magdalinou N., Lees A.J., Andreasson U., Norgren N., Linder J., Forsgren L., Constantinescu R. (2017). Blood-based NfL: A biomarker for differential diagnosis of Parkinsonian disorder. Neurology.

[104] Parnetti L., Gaetani L., Eusebi P., Paciotti S., Hansson O., El-Agnaf O., Mollenhauer B., Blennow K., Calabresi P. (2019). CSF and blood biomarkers for Parkinson’s disease. Lancet Neurol..

[105] Hall S., Öhrfelt A., Constantinescu R., Andreasson U., Surova Y., Bostrom F., Nilsson C., Håkan W., Decraemer H., Någga K. (2012). Accuracy of a panel of 5 cerebrospinal fluid biomarkers in the differential diagnosis of patients with dementia and/or Parkinsonian disorders. Arch. Neurol..

[106] Aamodt W.W., Waligorska T., Shen J., Tropea T.F., Siderowf A., Weintraub D., Grossman M., Irwin D., Wolk D.A., Xie S.X. (2021). Neurofilament light chain as a biomarker for cognitive decline in Parkinson disease. Mov. Disord..

[107] Sheng Z.H., Ma L.Z., Liu J.Y., Ou Y.N., Zhao B., Ma Y.H., Tan L. (2022). Cerebrospinal fluid neurofilament dynamic profiles predict cognitive progression in individuals with de novo Parkinson’s disease. Front. Aging Neurosci..

[108] Buhmann C., Lezius S., Pötter-Nerger M., Gerloff C., Kuhle J., Choe C.U. (2022). Age-adjusted serum neurofilament predicts cognitive decline in Parkinson’s disease (MARK-PD). Mov. Disord..

[109] Ma L.Z., Zhang C., Wang H., Ma Y.H., Shen X.N., Wang J., Tan L., Dong Q., Yu J.T. (2021). Serum neurofilament dynamics predicts cognitive progression in de novo Parkinson’s disease. J. Parkinson’s Dis..

[110] Gibson L.L., Pollak T.A., Heslegrave A., Hye A., Batzu L., Rota S., Trivedi D., Nicholson T.R., Ffytche D., Zetterberg H. (2022). Plasma neurofilament light and P-Tau181 and risk of psychosis in Parkinson’s disease. J. Parkinson’s Dis..

[111] Berciano J., García A., Gallardo E., Peeters K., Pelayo-Negro A.L., Álvarez-Paradelo S., Gazulla J., Martínez-Tames M., Infante J., Jordanova A. (2017). Intermediate Charcot-Marie-Tooth disease: An electrophysiological reappraisal and systematic review. J. Neurol..

[112] Morena J., Gupta A., Hoyle J.C. (2019). Charcot-Marie-Tooth: From molecules to therapy. Int. J. Mol. Sci..

[113] Gentil B.J., Minotti S., Beange M., Baloh R.H., Julien J., Durham H.D. (2012). Normal role of the low-molecular-weight neurofilament protein in mitochondrial dynamics and disruption in Charcot-Marie-Tooth disease. FASEB J..

[114] Zhao J., Brown K., Liem R.K.H. (2017). Abnormal neurofilament inclusions and segregations in dorsal root ganglia of a Charcot-Marie-Tooth type 2E mouse model. PLoS ONE.

[115] Yum S.W., Zhang J., Mo K., Li J., Scherer S.S. (2009). A novel recessive Nefl mutation causes a severe, early-onset axonal neuropathy. Ann. Neurol..

[116] Stone E.J., Kolb S.J., Brown A. (2021). A review and analysis of the clinical literature on Charcot-Marie-Tooth disease caused by mutations in neurofilament protein L.. Cytoskeleton.

[117] Brownlees J., Ackerley S., Grierson A.J., Jacobsen N.J.O., Shea K., Anderton B.H., Leigh P.N., Shaw C.E., Miller C.C.J. (2002). Charcot-Marie-Tooth disease neurofilament mutations disrupt neurofilament assembly and axonal transport. Hum. Mol. Genet..

[118] Sasaki T., Gotow T., Shiozaki M., Sakaue F., Saito T., Julien J.P., Uchiyama Y., Hisanaga S.I. (2006). Aggregate formation and phosphorylation of neurofilament-L Pro22 Charcot-Marie-Tooth disease mutants. Hum. Mol. Genet..

[119] Huynh D.T., Tsolova K.N., Watson A.J., Khal S.K., Green J.R., Li D., Hu J., Soderblom E.J., Chi J.T., Evans C.S. (2023). O-GlcNAcylation regulates neurofilament-light assembly and function and is perturbed by Charcot-Marie-Tooth disease mutations. Nat. Commun..

[120] Abe A., Numakura C., Saito K., Koide H., Oka N., Honma A., Kishikawa Y., Hayasaka K. (2009). Neurofilament light chain polypeptide gene mutations in Charcot-Marie-Tooth disease: Nonsense mutation probably causes a recessive phenotype. J. Hum. Genet..

[121] Horga A., Laurà M., Jaunmuktane Z., Jerath N.U., Gonzalez M.A., Polke J.M., Poh R., Blake J.C., Liu Y.T., Wiethoff S. (2017). Genetic and clinical characteristics of NEFL-related Charcot-Marie-Tooth disease. J. Neurol. Neurosurg. Psychiatry.

[122] Saveri P., De Luca M., Nisi V., Pisciotta C., Romano R., Piscosquito G., Reilly M.M., Polke J.M., Cavallaro T., Maria Fabrizi G. (2020). Charcot-Marie-Tooth Type 2B: A new phenotype associated with a novel RAB7A mutation and inhibited EGFR degradation. Cells.

[123] Setlere S., Grosmane A., Kurjane N., Gailite L., Rots D., Blennow K., Zetterberg H., Kenina V. (2023). Plasma neurofilament light chain level is not a biomarker of Charcot-Marie-Tooth disease progression: Results of 3-year follow-up study. Eur. J. Neurol..

[124] Ahmed R.E., Tokuyama T., Anzai T., Chanthra N., Uosaki H. (2022). Sarcomere maturation: Function acquisition, molecular mechanism, and interplay with other organelles. Philos. Trans. R. Soc. Lond. B Biol. Sci..

[125] Granger B.L., Lazarides E. (1979). Desmin and vimentin coexist at the periphery of the myofibril Z disc. Cell.

[126] Gard D.L., Lazarides E. (1980). The synthesis and distribution of desmin and vimentin during myogenesis in vitro. Cell.

[127] Tokuyasu K.T., Maher P.A., Singer S.J. (1985). Distributions of vimentin and desmin in developing chick myotubes in vivo. II. Immunoelectron microscopic study. J. Cell Biol..

[128] Solomon T., Rajendran M., Rostovtseva T., Hool L. (2022). How cytoskeletal proteins regulate mitochondrial energetics in cell physiology and diseases. Philos. Trans. R. Soc. Lond. B Biol. Sci..

[129] Fernández Casafuz A.B., De Rossi M.C., Bruno L. (2023). Mitochondrial cellular organization and shape fluctuations are differentially modulated by cytoskeletal networks. Sci. Rep..

[130] Eibauer M., Weber M.S., Kronenberg-Tenga R., Beales C.T., Boujemaa-Paterski R., Turgay Y., Sivagurunathan S., Kraxner J., Köster S., Goldman R.D. (2024). Vimentin filaments integrate low-complexity domains in a complex helical structure. Nat. Struct. Mol. Biol..

[131] Pérez-Sala D., Oeste C.L., Martínez A.E., Carrasco M.J., Garzón B., Cañada F.J. (2015). Vimentin filament organization and stress sensing depend on its single cysteine residue and zinc binding. Nat. Commun..

[132] Viedma-Poyatos Á., de Pablo Y., Pekny M., Pérez-Sala D. (2018). The cysteine residue of glial fibrillary acidic protein is a critical target for lipoxidation and required for efficient network organization. Free Radic. Biol. Med..

[133] Moneo-Corcuera D., Viedma-Poyatos Á., Stamatakis K., Pérez-Sala D. (2023). Desmin Reorganization by stimuli inducing oxidative stress and electrophiles: Role of its single cysteine residue. Antioxidants.

[134] Quinlan R.A., Franke W.W. (1982). Heteropolymer filaments of vimentin and desmin in vascular smooth muscle tissue and cultured baby hamster kidney cells demonstrated by chemical crosslinking. Proc. Natl. Acad. Sci. USA.

[135] Quinlan R.A., Franke W.W. (1983). Molecular interactions in intermediate-sized filaments revealed by chemical cross-linking. heteropolymers of vimentin and glial filament protein in cultured human glioma cells. Eur. J. Biochem..

[136] Kaus-Drobek M., Mücke N., Szczepanowski R.H., Wedig T., Czarnocki-Cieciura M., Polakowska M., Herrmann H., Wysłouch-Cieszyńska A., Dadlez M. (2020). Vimentin S-glutathionylation at Cys328 inhibits filament elongation and induces severing of mature filaments in vitro. FEBS J..

[137] Mónico A., Duarte S., Pajares M.A., Pérez-Sala D. (2019). Vimentin disruption by lipoxidation and electrophiles: Role of the cysteine residue and filament dynamics. Redox Biol..

[138] Rogers K.R., Herrmann H., Franke W.W. (1996). Characterization of disulfide crosslink formation of human vimentin at the dimer, tetramer, and intermediate filament levels. J. Struct. Biol..

[139] Day N.J., Kelly S.S., Lui L.Y., Mansfield T.A., Gaffrey M.J., Trejo J.B., Sagendorf T.J., Attah I.K., Moore R.J., Douglas C.M. (2024). Signatures of cysteine oxidation on muscle structural and contractile proteins are associated with physical performance and muscle function in older adults: Study of muscle, mobility and aging (SOMMA). Aging Cell.

[140] Dutka T.L., Mollica J.P., Lamboley C.R., Weerakkody V.C., Greening D.W., Posterino G.S., Murphy R.M., Lamb G.D. (2017). S-nitrosylation and S-glutathionylation of Cys134 on troponin I have opposing competitive actions on Ca^2+^ sensitivity in rat fast-twitch muscle fibers. Am. J. Physiol. Cell Physiol..

[141] Giganti D., Yan K., Badilla C.L., Fernandez J.M., Alegre-Cebollada J. (2018). Disulfide isomerization reactions in titin immunoglobulin domains enable a mode of protein elasticity. Nat. Commun..

[142] Matsui R., Ferran B., Oh A., Croteau D., Shao D., Han J., Pimentel D.R., Bachschmid M.M. (2020). Redox regulation via glutaredoxin-1 and protein S-glutathionylation. Antioxid. Redox Signal..

[143] Alcock L.J., Perkins M.V., Chalker J.M. (2018). Chemical methods for mapping cysteine oxidation. Chem. Soc. Rev..

[144] Devarie Baez N.O., Reisz J.A., Furdui C.M. (2015). Mass spectrometry in studies of protein thiol chemistry and signaling: Opportunities and caveats. Free Radic. Biol. Med..

[145] Shi Y., Carroll K.S. (2020). Activity-based sensing for site-specific proteomic analysis of cysteine oxidation. Acc. Chem. Res..

[146] González-Jiménez P., Duarte S., Martínez A.E., Navarro-Carrasco E., Lalioti V., Pajares M.A., Pérez-Sala D. (2023). Vimentin single cysteine residue acts as a tunable sensor for network organization and as a key for actin remodeling in response to oxidants and electrophiles. Redox Biol..

[147] Pekovic V., Gibbs-Seymour I., Markiewicz E., Alzoghaibi F., Benham A.M., Edwards R., Wenhert M., von Zglinicki T., Hutchison C.J. (2011). Conserved cysteine residues in the mammalian lamin a tail are essential for cellular responses to ROS generation. Aging Cell.

[148] Unoki T., Akiyama M., Kumagai Y. (2020). Nrf2 activation and its coordination with the protective defense systems in response to electrophilic stress. Int. J. Mol. Sci..

[149] Marzetti E., Calvani R., Landi F., Coelho-Júnior H.J., Picca A. (2024). Mitochondrial quality control processes at the crossroads of cell death and survival: Mechanisms and signaling pathways. Int. J. Mol. Sci..

[150] Picca A., Mankowski R.T., Burman J.L., Donisi L., Kim J.-S.S., Marzetti E., Leeuwenburgh C. (2018). Mitochondrial quality control mechanisms as molecular targets in cardiac ageing. Nat. Rev. Cardiol..

[151] Picca A., Faitg J., Auwerx J., Ferrucci L., D’Amico D. (2023). Mitophagy in human health, ageing and disease. Nat. Metab..

[152] Anesti V., Scorrano L. (2006). The relationship between mitochondrial shape and function and the cytoskeleton. Biochim. Biophys. Acta.

[153] Moore A.S., Holzbaur E.L. (2018). Mitochondrial-cytoskeletal interactions: Dynamic associations that facilitate network function and remodeling. Curr. Opin. Physiol..

[154] Kurd D.D., Saxton W.M. (1996). Kinesin mutations cause motor neuron disease phenotypes by disrupting fast axonal transport in drosophila. Genetics.

[155] Pilling A.D., Horiuchi D., Lively C.M., Saxton W.M. (2006). Kinesin-1 and dynein are the primary motors for fast transport of mitochondria in Drosophila motor axons. Mol. Biol. Cell.

[156] Guo X., Macleod G.T., Wellington A., Hu F., Panchumarthi S., Schoenfield M., Marin L., Charlton M.P., Atwood H.L., Zinsmaier K.E. (2005). The GTPase DMiro is required for axonal transport of mitochondria to Drosophila synapses. Neuron.

[157] Stowers R.S., Megeath L.J., Górska-Andrzejak J., Meinertzhagen I.A., Schwarz T.L. (2002). Axonal transport of mitochondria to synapses depends on Milton, a novel Drosophila protein. Neuron.

[158] Cardanho-Ramos C., Faria-Pereira A., Morais V.A. (2020). Orchestrating mitochondria in neurons: Cytoskeleton as the conductor. Cytoskeleton.

[159] Lezi E., Swerdlow R.H. (2012). Mitochondria in neurodegeneration. Adv. Exp. Med. Biol..

[160] Alberti P., Semperboni S., Cavaletti G., Scuteri A. (2022). Neurons: The interplay between cytoskeleton, ion channels/transporters and mitochondria. Cells.

[161] Campbell P.D., Shen K., Sapio M.R., Glenn T.D., Talbot W.S., Marlow F.L. (2014). Unique function of kinesin Kif5A in localization of mitochondria in axons. J. Neurosci..

[162] Guillaud L., El-Agamy S.E., Otsuki M., Terenzio M. (2020). Anterograde axonal transport in neuronal homeostasis and disease. Front. Mol. Neurosci..

[163] Brenner D., Yilmaz R., Müller K., Grehl T., Petri S., Meyer T., Grosskreutz J., Weydt P., Ruf W., Neuwirth C. (2018). Hot-Spot KIF5A mutations cause familial ALS. Brain.

[164] Saez-Atienzar S., Dalgard C.L., Ding J., Chiò A., Alba C., Hupalo D.N., Wilkerson M.D., Bowser R., Pioro E.P., Bedlack R. (2020). Identification of a pathogenic intronic KIF5A mutation in an ALS-FTD kindred. Neurology.

[165] Filosto M., Piccinelli S.C., Palmieri I., Necchini N., Valente M., Zanella I., Biasiotto G., Di Lorenzo D., Cereda C., Padovani A. (2018). A novel mutation in the stalk domain of KIF5A causes a slowly progressive atypical motor syndrome. J. Clin. Med..

[166] Nicolas A., Kenna K., Renton A.E., Ticozzi N., Faghri F., Chia R., Dominov J.A., Kenna B.J., Nalls M.A., Keagle P. (2018). Genome-wide analyses identify KIF5A as a novel ALS Gene. Neuron.

[167] Zhao J., Wang Y., Xu H., Fu Y., Qian T., Bo D., Lu Y.X., Xiong Y., Wan J., Zhang X. (2016). Dync1h1 Mutation causes proprioceptive sensory neuron loss and impaired retrograde axonal transport of dorsal root ganglion neurons. CNS Neurosci. Ther..

[168] Chen X.J., Levedakou E.N., Millen K.J., Wollmann R.L., Soliven B., Popko B. (2007). Proprioceptive sensory neuropathy in mice with a mutation in the cytoplasmic dynein heavy chain 1 gene. J. Neurosci..

[169] Weedon M.N., Hastings R., Caswell R., Xie W., Paszkiewicz K., Antoniadi T., Williams M., King C., Greenhalgh L., Newbury-Ecob R. (2011). Exome sequencing identifies a DYNC1H1 mutation in a large pedigree with dominant axonal Charcot-Marie-Tooth disease. Am. J. Hum. Genet..

[170] Theunissen F., West P.K., Brennan S., Petrović B., Hooshmand K., Akkari P.A., Keon M., Guennewig B. (2021). New perspectives on cytoskeletal dysregulation and mitochondrial mislocalization in amyotrophic lateral sclerosis. Transl. Neurodegener..

[171] Hsieh C., Shaltouki A., Gonzalez A., Bettencourt da Cruz A., Burbulla L., St Lawrence E., Schüle B., Krainc D., Palmer T., Wang X. (2016). Functional impairment in Miro degradation and mitophagy is a shared feature in familial and sporadic Parkinson’s disease. Cell Stem Cell.

[172] Knippenberg S., Sipos J., Thau-Habermann N., Körner S., Rath K.J., Dengler R., Petri S. (2013). Altered expression of DJ-1 and PINK1 in sporadic ALS and in the SOD1(G93A) ALS mouse model. J. Neuropathol. Exp. Neurol..

[173] Sun X., Duan Y., Qin C., Li J.C., Duan G., Deng X., Ni J., Cao X., Xiang K., Tian K. (2018). Distinct multilevel misregulations of Parkin and PINK1 revealed in cell and animal models of TDP-43 proteinopathy. Cell Death Dis..

[174] Chen Y., Deng J., Wang P., Yang M., Chen X., Zhu L., Liu J., Lu B., Shen Y., Fushimi K. (2016). PINK1 and Parkin are genetic modifiers for FUS-induced neurodegeneration. Hum. Mol. Genet..

[175] Wu Y., Ding C., Sharif B., Weinreb A., Swaim G., Hao H., Yogev S., Watanabe S., Hammarlund M. (2024). Polarized localization of kinesin-1 and RIC-7 drives axonal mitochondria anterograde transport. J. Cell Biol..

[176] Van Steenbergen V., Lavoie-Cardinal F., Kazwiny Y., Decet M., Martens T., Verstreken P., Boesmans W., De Koninck P., Vanden Berghe P. (2022). Nano-positioning and tubulin conformation contribute to axonal transport regulation of mitochondria along microtubules. Proc. Natl. Acad. Sci. USA.

[177] Manor U., Bartholomew S., Golani G., Christenson E., Kozlov M., Higgs H., Spudich J., Lippincott-Schwartz J. (2015). A Mitochondria-anchored isoform of the actin-nucleating spire protein regulates mitochondrial division. eLife.

[178] Friedman J.R., Lackner L.L., West M., DiBenedetto J.R., Nunnari J., Voeltz G.K. (2011). ER tubules mark sites of mitochondrial division. Science.

[179] Smirnova E., Griparic L., Shurland D.L., Van der Bliek A.M. (2001). Dynamin-related protein Drp1 is required for mitochondrial division in mammalian cells. Mol. Biol. Cell.

[180] Chai N., Haney M.S., Couthouis J., Morgens D.W., Benjamin A., Wu K., Ousey J., Fang S., Finer S., Bassik M.C. (2020). Genome-wide synthetic lethal CRISPR screen identifies FIS1 as a genetic interactor of ALS-linked C9ORF72. Brain Res..

[181] Shen Q., Yamano K., Head B.P., Kawajiri S., Cheung J.T.M., Wang C., Cho J.H., Hattori N., Youle R.J., Van Der Bliek A.M. (2014). Mutations in Fis1 disrupt orderly disposal of defective mitochondria. Mol. Biol. Cell.

[182] Heissler S.M., Sellers J.R. (2016). Various themes of myosin regulation. J. Mol. Biol..

[183] Quintero O.A., DiVito M.M., Adikes R.C., Kortan M.B., Case L.B., Lier A.J., Panaretos N.S., Slater S.Q., Rengarajan M., Feliu M. (2009). Human Myo19 is a novel myosin that associates with mitochondria. Curr. Biol..

[184] Lu Z., Ma X.N., Zhang H.M., Ji H.H., Ding H., Zhang J., Luo D., Sun Y., Li X.D. (2014). Mouse myosin-19 is a plus-end-directed, high-duty ratio molecular motor. J. Biol. Chem..

[185] López-Doménech G., Covill-Cooke C., Ivankovic D., Halff E.F., Sheehan D.F., Norkett R., Birsa N., Kittler J.T. (2018). Miro proteins coordinate microtubule- and actin-dependent mitochondrial transport and distribution. EMBO J..

[186] Cipriani S., Guerrero-Valero M., Tozza S., Zhao E., Vollmer V., Beijer D., Danzi M., Rivellini C., Lazarevic D., Pipitone G.B. (2023). Mutations in MYO9B are associated with Charcot-Marie-Tooth disease type 2 neuropathies and isolated optic atrophy. Eur. J. Neurol..

